# Safety and Efficacy of a Recombinant Newcastle Disease Virus Expressing Cecropin AD for Diarrhea Reduction and Intestinal Health Improvement in Domestic Pigeons

**DOI:** 10.3390/ani16162454

**Published:** 2026-08-07

**Authors:** Lun Yao, Hongcai Wang, Longshun Li, Qianni Xiao, Zhe Zeng, Yu Shang, Helong Feng, Donghui Li, Mengyun Jin, Huabin Shao, Qingping Luo, Guoyuan Wen

**Affiliations:** 1 Key Laboratory of Prevention and Control Agents for Animal Bacteriosis, Ministry of Agriculture and Rural Affairs, Institute of Animal Sciences and Veterinary Medicine, Hubei Academy of Agricultural Sciences, Wuhan 430064, China; yaolun027@126.com (L.Y.); wanghongcai@hbaas.ac.cn (H.W.); xqn3215460@163.com (Q.X.); zengzhe@hbaas.ac.cn (Z.Z.); suppershangyu@126.com (Y.S.); fenghelong_clp@163.com (H.F.); 15037603756@163.com (M.J.); shhb1961@163.com (H.S.); 2 Key Laboratory of Animal Pathogenic Microbiology of Hubei Province, Institute of Animal Husbandry and Veterinary, Hubei Academy of Agricultural Sciences, Wuhan 430064, China; 3 Hubei Hongshan Laboratory, Wuhan 430064, China; 4 Hubei Agricultural Development Beidi Pigeon Technology Co., Ltd., Xiangyang 441000, China; nfbd6666099@163.com (L.L.); 15549962444@163.com (D.L.)

**Keywords:** diarrhea, CAD, NDV vector, intestinal health

## Abstract

Diarrhea and impaired intestinal development are major constraints in newly hatched domestic pigeons raised for meat production, leading to reduced growth performance and increased reliance on antimicrobial interventions. Cecropin AD (CAD) is a broad-spectrum antimicrobial peptide with potential activity against bacterial pathogens and may represent an alternative strategy for improving intestinal health in poultry. In this study, we evaluated the safety and biological effects of a recombinant Newcastle disease virus (NDV) expressing CAD (rTS-CAD_3_) in one-day-old domestic pigeons. Pigeons were inoculated with rTS-CAD_3_, parental TS09-C, or PBS and monitored for growth performance, diarrhea incidence, viral RNA persistence, inflammatory responses, intestinal bacterial abundance, gut microbiota composition, intestinal morphology, and epithelial barrier function. rTS-CAD_3_ caused no mortality, detected only transient viral RNA, and did not induce excessive inflammatory responses. Compared with TS09-C and PBS controls, rTS-CAD_3_ significantly increased body weight gain and average daily gain while reducing diarrhea incidence. Importantly, rTS-CAD_3_ decreased intestinal *Escherichia coli* abundance, reshaped the gut microbiota by reducing Proteobacteria and Escherichia–Shigella while increasing Firmicutes and Lactobacillus, improved villus height and the villus-to-crypt ratio, and enhanced occludin expression. These findings demonstrate that NDV-vectored CAD delivery can reduce intestinal bacterial burden, increase occludin expression, and alleviate diarrhea in domestic pigeons. The rTS-CAD_3_ represents a promising antimicrobial peptide-based strategy for reducing spontaneous diarrhea and dependence on conventional antibiotics in poultry production.

## Simple Summary

Diarrhea is a common problem in young domestic pigeons and can impair intestinal health and growth. Antibiotics are often used for treatment, but their use raises concerns about residues and antimicrobial resistance. Antimicrobial peptides are considered potential alternatives. In this study, we evaluated a recombinant Newcastle disease virus expressing the antimicrobial peptide cecropin AD, named rTS-CAD_3_, in newborn pigeons. rTS-CAD_3_ caused no mortality and showed only transient viral RNA detection. It reduced diarrhea incidence, decreased intestinal *Escherichia coli* abundance, and improved body weight gain. The treatment also altered gut microbiota composition, increased intestinal villus height and villus-to-crypt ratio, and enhanced occludin expression, suggesting improved intestinal barrier function. These findings indicate that NDV-vectored antimicrobial peptide delivery may be a useful strategy for improving gut health in pigeon production.

## 1. Introduction

Pigeon meat is highly nutritious, with high protein and low fat contents, and has attracted increasing consumer interest globally, with studies highlighting its quality traits and significance in production [1]. However, diarrhea remains a major constraint in squab production, especially during early life when intestinal development is immature and birds are vulnerable to bacterial overgrowth, gut dysbiosis, and environmental stress [2]. The current strategy for controlling diarrhea in pigeon farms often relies on antibiotics, raising concerns about residues in meat, given the short production cycle.

Antimicrobial peptides are bioactive molecules with broad-spectrum antibacterial activity and minimal toxicity, and represent promising alternatives to antibiotics [3]. Cecropin AD (CAD) is a hybrid antimicrobial peptide composed of cecropin A and D domains, and dietary CAD supplementation has been shown to alleviate *Escherichia coli*-induced diarrhea and improve intestinal health in piglets [4]. Recent studies have also demonstrated that cecropin peptides can enhance growth performance, reduce diarrhea rates, and positively modulate gut microbial composition in animal models [5]. However, it remains unclear whether cecropins can promote growth and modulate the intestinal flora in poultry, especially pigeons.

The Newcastle disease virus (NDV) is widely used as a vector for multivalent vaccine development in poultry and provides an effective platform for the expression of exogenous proteins [6]. Our previous study demonstrated that CAD-expressing NDV significantly reduced bacterial infections at murine skin wound sites and promoted wound healing in mice [7]. Here, we evaluated the safety of recombinant CAD-expressing NDV in newborn pigeons and investigated its effects on growth performance, intestinal morphology, tight junction expression, intestinal microbiota, and aimed to support the application of cecropins in pigeon production.

## 2. Materials and Methods

### 2.1. Pathogenicity Analysis and Virus Titration

The pathogenicity of rTS-CAD_3_, which was previously described by our laboratory, was evaluated by measuring the mean death time of the minimal lethal dose (MDT/MLD) in 10-day-old SPF embryonated chicken eggs [7]. MDT values greater than 90 h indicate low virulence, between 60 and 90 h indicate moderate virulence, and less than 60 h indicate high virulence. The 50% embryonic infectious dose (EID_50_) of the isolate was calculated using the Reed and Muench method in SPF embryonated chicken eggs.

### 2.2. Safety Assessment in Newborn Pigeons

For the safety assessment, a total of 150 newborn white hybrid meat pigeons were allocated to three groups (50 pigeons per group) with similar body weights (mean initial body weight was 19.0 ± 1.0 g). The pigeons were inoculated with rTS-CAD_3_ or TS09-C (10^6.0^ EID_50_/mL, 50 μL per pigeon) via the intranasal route. PBS was used as a control. Each pair of newly hatched squabs was reared using a single parent pigeon pair. All parent pigeons were 3 years old, and no significant differences in feeding capacity or health status were observed prior to the experiment. The experiment lasted 21 days. During the test period, six pigeons squabs per group were humanely euthanized every 3 days, and lung and intestinal tissues were collected to assess viral colonization. Squabs from the same parental pair were assigned to the same/different treatment groups. Blood samples were collected at 0, 3, 6, and 9 days post-infection (dpi) for cytokine expression analyses.

### 2.3. Quantification of NDV Load in Lung and Duodenum

Lung and duodenum tissues were collected at 0, 3, 6, 9, 12, 15, 18, and 21 dpi for viral detection using a quantitative real-time PCR (qPCR) assay. Total RNA was extracted (0.5 g tissue), quantified, and reverse transcribed (1 μg RNA). qPCR was performed to determine NDV load based on a standard curve of the NDV F gene. NDV F gene primer sequences: forward 5′-ATGGGCTCCAGATCTTCTAC-3′; reverse 5′-TCCTACGGATAGAATCACC-3′.

### 2.4. Cytokine Expression Analysis

Blood (100 μL) collected from six pigeons per group was centrifuged (1500× *g*, 10 min, 4 °C; then 2000× *g*, 15 min, 4 °C) to obtain platelet-depleted plasma. IL-6 and granulocyte colony-stimulating factor (G-CSF) levels were quantified by ELISA (Cusabio, Wuhan, China) following the manufacturer’s protocol.

### 2.5. Growth Performance, Diarrhea Index and Sample Collection

For the efficacy assessment, an independent cohort of 150 newborn white hybrid meat pigeons was inoculated with rTS-CAD_3_ (10^6.0^ EID_50_/mL, 50 μL per bird) via the intranasal route as described in the safety assessment assay. During the experiment, all pigeons were weighed every 3 days and recorded repeatedly. The average body weight (BW), average daily gain (ADG), and survival rate were measured and calculated for each repetition at 21 days of age. Fecal consistency was evaluated once daily by one observer blinded to the treatment groups; therefore, inter-observer disagreement was not applicable. Diarrhea index was scored 0–3 (0: normal solid; 1: moist semi-solid; 2: mild loose; 3: severe watery). Scores ≥ 2 were considered diarrhea [8]. Colonic contents were collected at 21 dpi using sterile cryopreservation tubes and quickly frozen in liquid nitrogen. The duodenum and jejunum were collected at 21 dpi for histological analysis. The intestinal contents were collected at 0, 6, 12, and 18 dpi for *E. coli* enumeration.

### 2.6. Assessment of Gut Microbial Diversity

Three samples of colonic content from each group were used for high-throughput sequencing, as previously reported [9]. DNA was extracted (cetyltrimethylammonium bromide method), and the V3–V4 region was amplified with primers 341F/806R, followed by sequencing on an Illumina NovaSeq 6000 platform (PE250) (Illumina, Inc., San Diego, CA, USA). Sequence processing, ASV inference, taxonomic annotation, and diversity analyses were performed using QIIME2 (2024.2), R (4.5.1), and LEfSe (1.1.2).

### 2.7. Small Intestinal Morphometric Traits

Approximately 1 cm segments of the duodenum and jejunum were collected at 21 dpi, gently rinsed with physiological saline, and fixed in 4% formalin for 24 h. The tissues were subsequently dehydrated, paraffin-embedded, sectioned, then deparaffinized in xylene and rehydrated in graded ethanol. For each bird, six randomly selected microscopic fields were examined using a BX53 microscope (Olympus, Tokyo, Japan). Correctly oriented villus-crypt units were selected when the entire villus axis, from the villus tip to the associated crypt opening, was visible without obvious folding or oblique sectioning. Villus height was measured from the villus tip to the villus–crypt junction, whereas crypt depth was measured from the crypt opening to its base. The villus-height-to-crypt-depth ratio was subsequently calculated.

### 2.8. Culture-Based Enumeration of Escherichia coli (E. coli)

The serially diluted intestinal contents, collected at 0, 6, 12, and 18 dpi, were plated on MacConkey agar. Following incubation at 37 °C for 24 h, lactose-fermenting colonies were counted, with representative colonies confirmed by Gram staining and PCR. The *E. coli* counts were expressed as log_10_ CFU/g of intestinal contents.

### 2.9. Immunohistochemistry (IHC)

At 21 dpc, six pigeons per group were randomly selected for tissue collection, and the duodenal and jejunal tissues were sectioned, deparaffinized, peroxidase-blocked (0.3% H_2_O_2_), antigen-retrieved (Tris–EDTA, pH 9.0), serum-blocked, and incubated overnight at 4 °C with occludin polyclonal antibody (1:200, catalogue no. 71-1500, Thermo Fisher Scientific, Waltham, MA, USA). Immunoreactivity was visualized using a horseradish peroxidase-conjugated detection system with DAB as chromogen, followed by hematoxylin counterstaining. For each bird, six randomly selected non-overlapping microscopic fields per intestinal segment were captured at ×400 magnification using a digital camera (BX53; Olympus, Tokyo, Japan). Protein levels were semi-quantified using Image-Pro Plus software (6.2), with results expressed as the integrated optical density (IOD) of the positive area in microphotographs captured at ×400 magnification. A uniform staining threshold was established based on background staining and applied consistently to all images.

### 2.10. Quantitative Real-Time PCR Analysis

RNA from duodenal and jejunal tissues (six randomly selected pigeons/group at 21 dpi) was reverse transcribed to cDNA. For occludin mRNA expression, qPCR was performed using a SYBR Green I kit (Vazyme, Nanjing, China) with the following protocol: initial denaturation at 94 °C for 30 s; 40 cycles of 94 °C for 5 s and 62 °C for 30 s; melting curve from 55 °C to 95 °C (increment 0.5 °C/10 s). Relative occludin expression was calculated using the 2^−ΔΔCt^ method using GAPDH as the internal control. Occludin primer sequences: forward 5′-CAGGACGTGGCAGAGGA-3′; reverse 5′-GTGGAAGAGCTTGTTGCGT-3′.

### 2.11. Statistical Analysis

Statistical analyses were performed using GraphPadPrism 10. Repeatedly measured body weight and diarrhea scores were analyzed using a two-way repeated-measures ANOVA or a mixed-effects model, with treatment, time, and their interaction as fixed effects and individual pigeon as the repeated subject. Viral RNA loads, cytokine levels, and E. coli counts obtained from independently sampled pigeons at different time points were analyzed using two-way ANOVA, followed by Tukey’s multiple-comparison test. Endpoint data were analyzed using one-way ANOVA followed by Tukey’s test.

## 3. Results

### 3.1. Safety Assessment of rTS-CAD3 in Newborn Pigeons

**Figure 1:**
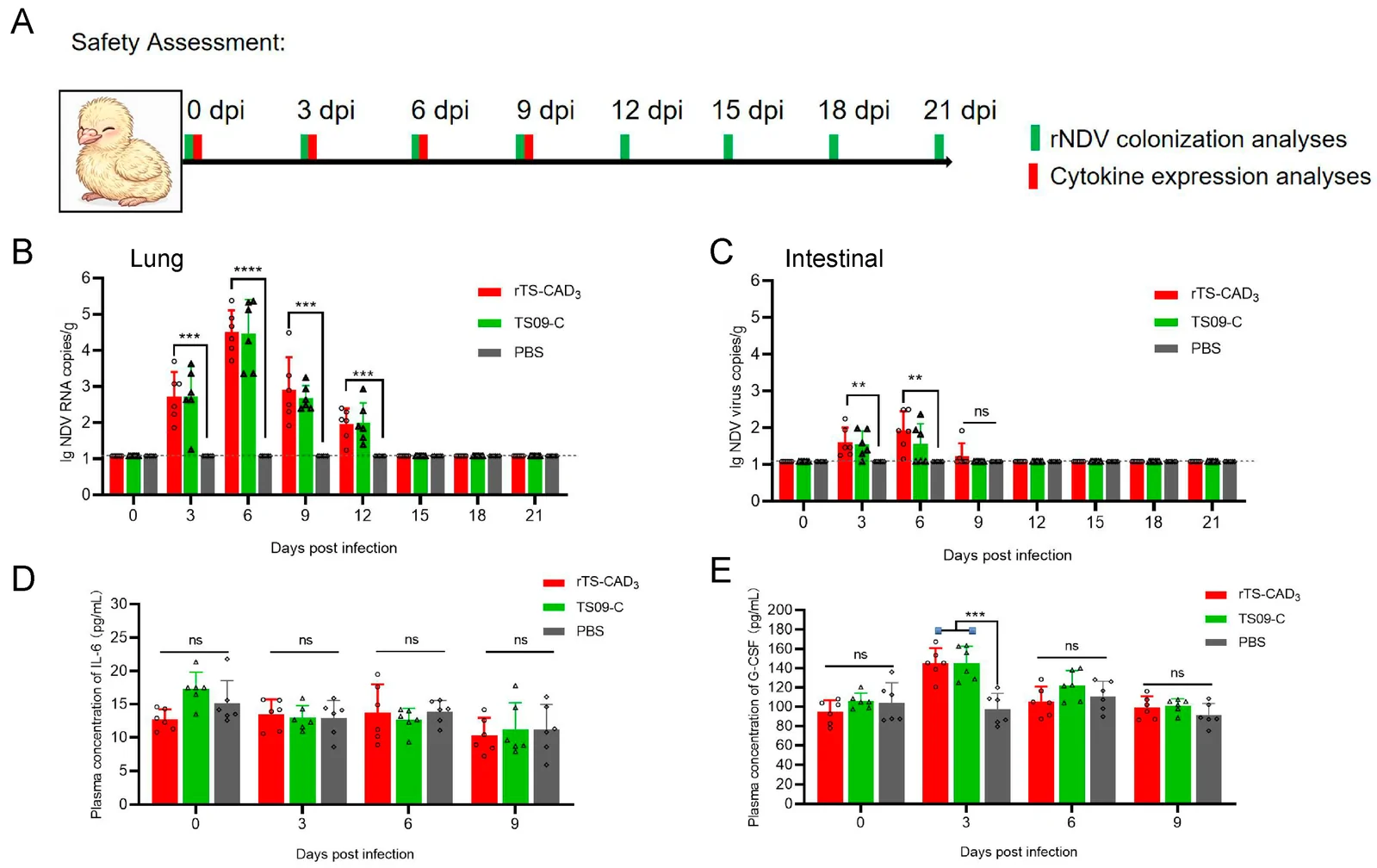
Safety evaluation of rTS-CAD_3_ in newborn pigeons in experimental conditions. (**A**) Experimental design and sampling timeline for safety evaluation. (**B**,**C**) NDV genomic RNA loads in lung tissues and duodenal tissues at the indicated time points were detected by qPCR. (**D**,**E**) Plasma concentrations of IL-6 and G-CSF. (**, *p* < 0.01; ***, *p* < 0.001; ****, *p* < 0.0001; ns, not significant).

To evaluate the safety of rTS-CAD_3_, rNDV-infected pigeons were monitored for 3 weeks. No mortality was observed in the rNDV-inoculated groups. The experimental design and sampling schedule for the safety assessment are shown in Figure 1A. Viral genomic RNA was transiently detected in the lungs from 3 to 12 dpi and in the duodenum tissues during the first 6 dpi, but became undetectable thereafter (Figure 1B,C). IL-6 levels showed no significant difference between the rNDV-inoculated and control groups, whereas G-CSF levels increased mildly at 3 dpi and returned to baseline by 6 dpi (Figure 1D,E), indicating that rNDVs did not induce excessive inflammatory responses. rTS-CAD_3_ retained the low pathogenicity of the parental TS09-C strain, with an MDT of >168 h (Table 1). These results suggest that rTS-CAD_3_ is safe for newborn pigeons in experimental conditions.

### 3.2. Growth Performance Assessment of rTS-CAD3 in Newborn Pigeons

**Figure 2:**
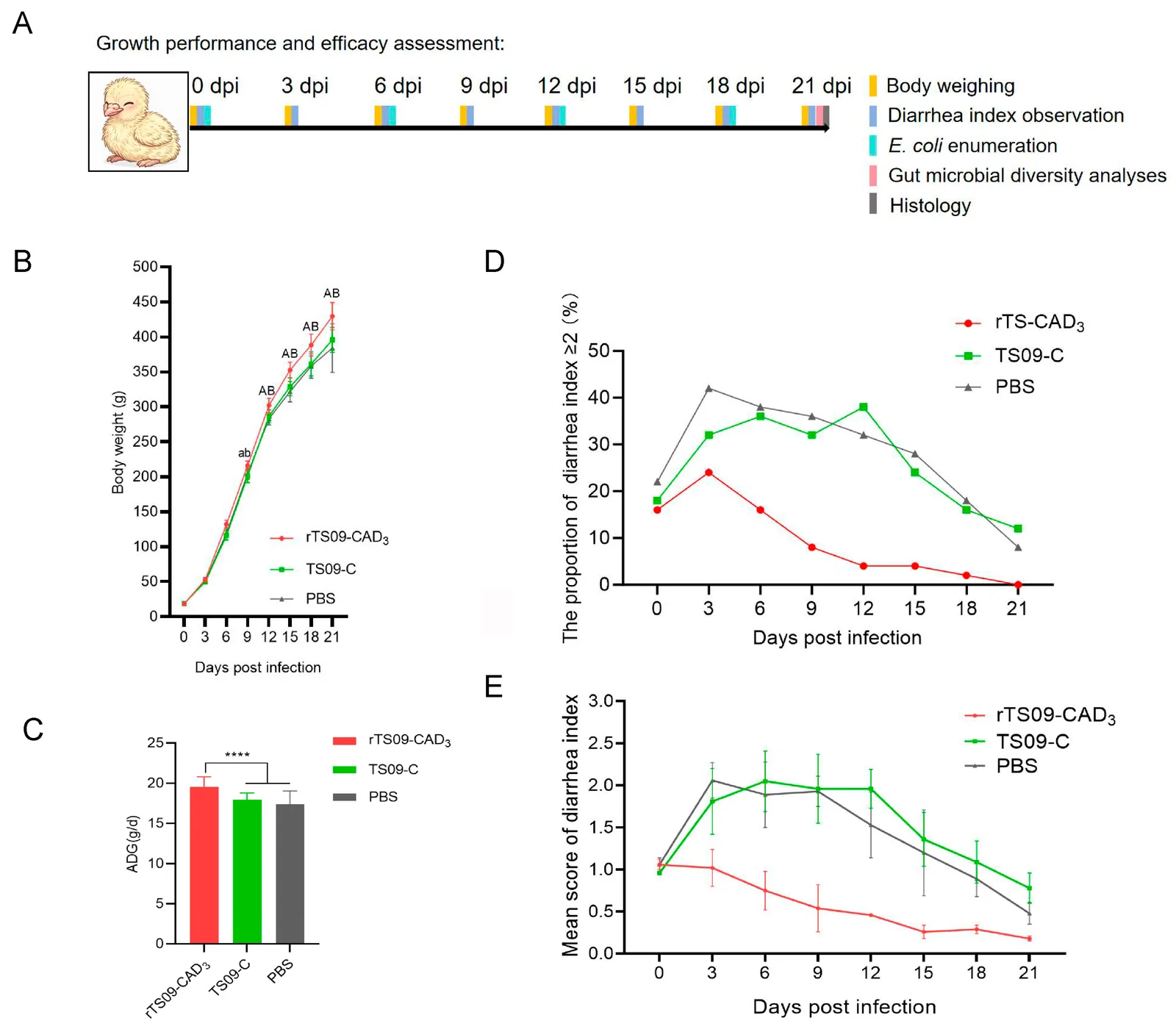
Growth performance and antidiarrheal effects of rTS-CAD_3_ in newborn pigeons. (**A**) Experimental design and sampling timeline for growth performance and efficacy assessment. (**B**) Body weight changes in pigeons during the experimental period. (**C**) ADG of pigeons. (**D**) The proportion of pigeons with a diarrhea score ≥ 2. (**E**) Mean diarrhea score of pigeons. (rTS-CAD_3_ vs. TS09-C, a, *p* < 0.05; A, *p* < 0.01; rTS-CAD_3_ vs. PBS, b, *p* < 0.05; B, *p* < 0.01). ****, *p* < 0.0001.

The experimental design and observation schedule for growth performance and efficacy assessment are shown in Figure 2A. All pigeons showed progressive body weight gain during the experiment. Compared with the TS09-C and PBS groups, pigeons treated with rTS-CAD_3_ exhibited higher body weights during the later stages of the experiment, with significant differences observed at several time points from 12 to 21 dpi (Figure 2B). Consistently, the average daily gain of the rTS-CAD_3_ group was significantly higher than that of the control groups (Figure 2C). The proportion of pigeons with a diarrhea score ≥ 2 decreased in the rTS-CAD_3_ group after 3 dpi and reached 0% at 21 dpi. In contrast, the TS09-C and PBS groups showed higher proportions of diarrhea-positive pigeons (Figure 2D). The mean diarrhea score remained relatively low in the rTS-CAD_3_ group throughout the observation period (Figure 2E). These results indicate that rTS-CAD_3_ improved growth performance and reduced diarrhea severity in newborn pigeons.

### 3.3. Effects of rTS-CAD_3_ on Intestinal Microbial Composition in Pigeons

**Figure 3:**
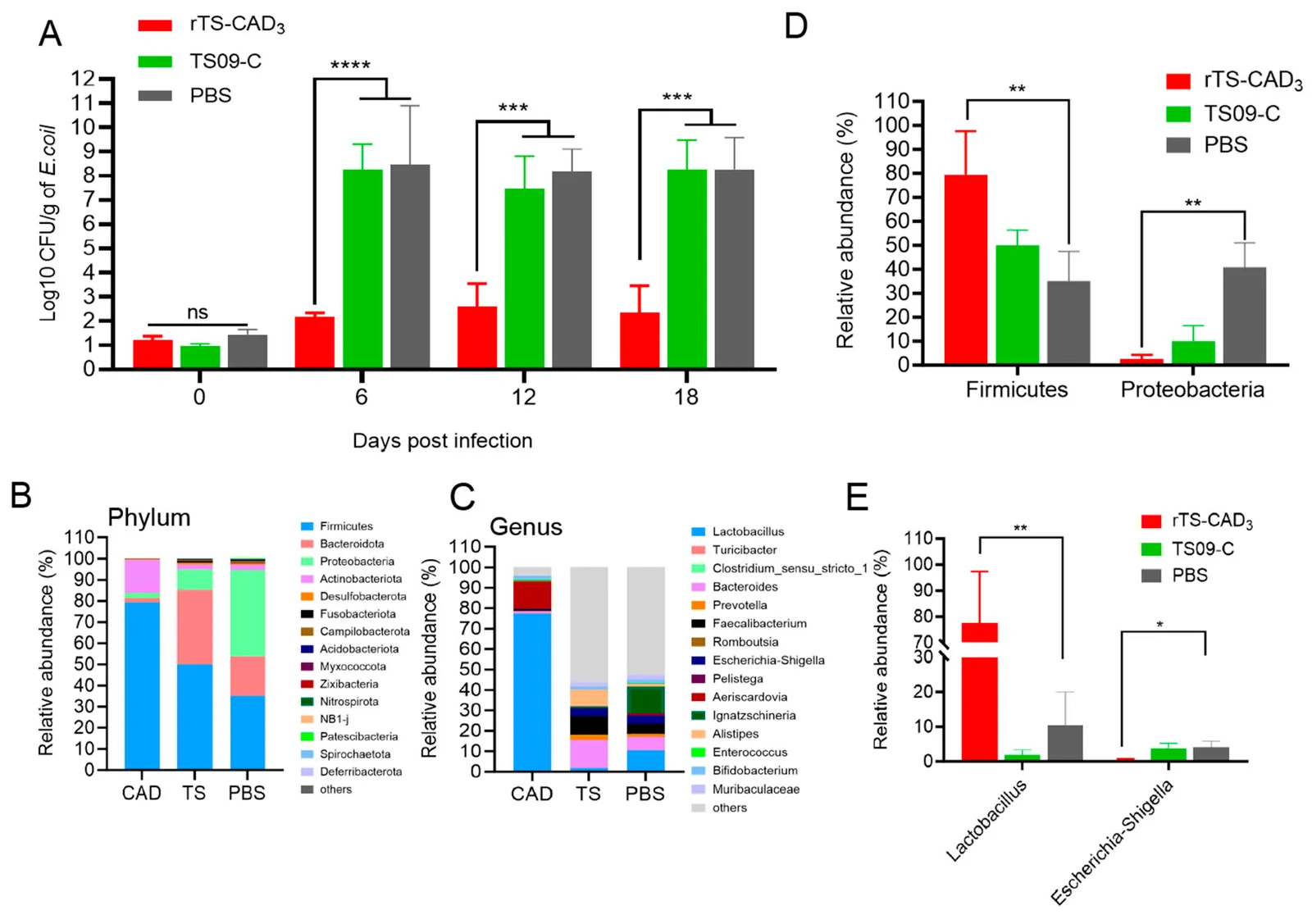
Effects of rTS-CAD_3_ on intestinal microbiota composition in pigeons. (**A**) *E. coli* contents at indicated time points, expressed as log_10_ CFU/g. (**B**,**C**) Relative bacterial abundance at the phylum and genus levels. CAD, rTS-CAD_3_ group. TS, TS09-C group. (**D**,**E**) Comparison of the relative abundance of Firmicutes and Proteobacteria at the phylum level, and the relative abundance of Lactobacillus and Escherichia–Shigella at the genus level among treatment groups. *, *p* < 0.05; **, *p* < 0.01; ***, *p* < 0.001; ****, *p* < 0.0001.

As rTS-CAD_3_ showed inhibitory activity against *E. coli* in our previous study [7], intestinal *E. coli* abundance was examined. Compared with the TS09-C and PBS groups, rTS-CAD_3_ significantly reduced intestinal *E. coli* levels at 6, 12, and 18 dpi (Figure 3A). In addition, 16S rRNA gene sequencing showed that rTS-CAD_3_ altered the intestinal microbial community. At the phylum level, rTS-CAD_3_-treated pigeons showed increased Firmicutes and decreased Proteobacteria, while at the genus level, the relative abundance of Escherichia–Shigella was significantly reduced, and Lactobacillus was significantly increased (Figure 3B–E). These changes indicate that rTS-CAD_3_ may improve intestinal microbial balance by suppressing enteric bacteria associated with dysbiosis and diarrhea.

### 3.4. Effects of rTS-CAD_3_ on Intestinal Morphology and Occludin Expression in Pigeons

**Figure 4:**
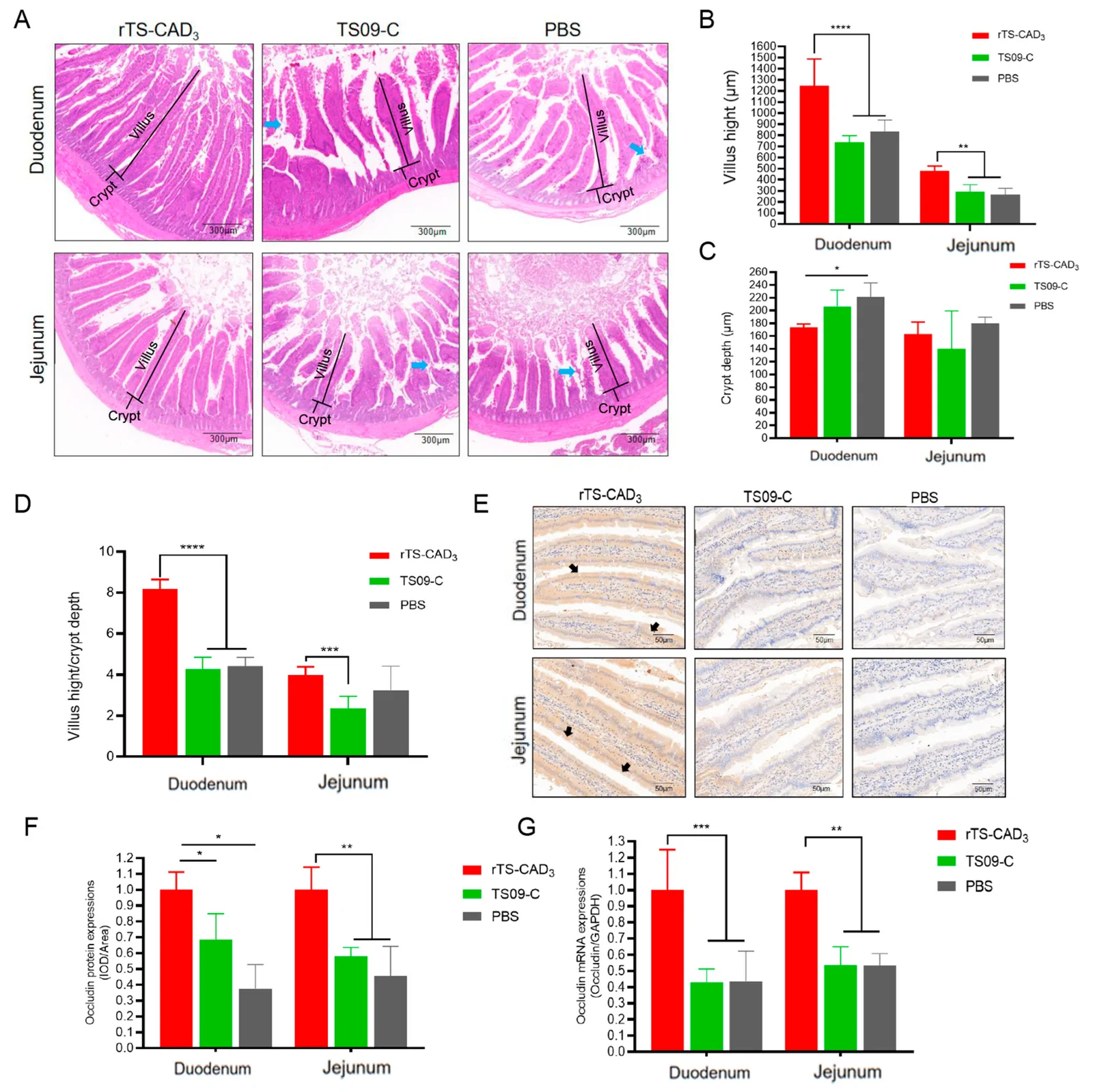
Effects of rTS-CAD_3_ on intestinal morphology and occludin expression in pigeons. (**A**) Representative H&E staining images of the duodenum and jejunum. Black lines schematically indicate villus height and crypt depth. Blue arrows indicate the shortened or irregular villi in the TS09-C and PBS groups. Scale bar = 300 μm. (**B**) Villus height of the duodenum and jejunum. (**C**) Crypt depth of the duodenum and jejunum. (**D**) Villus height-to-crypt depth (V/C) ratio. (**E**) Representative IHC staining of occludin protein in the duodenum and jejunum (scale bar = 50 μm). Black arrows indicate pronounced occludin-positive staining. (**F**) Semi-quantitative analysis of occludin protein expression based on IOD. (**G**) Relative mRNA levels of occludin in duodenum and jejunum tissues which were determined by using qPCR. *, *p* < 0.05; **, *p* < 0.01; ***, *p* < 0.001; ****, *p* < 0.0001.

Histological analysis showed that rTS-CAD_3_ significantly increased villus height in the duodenum and jejunum (Figure 4A,B). Although crypt depth increased in the duodenum and remained unchanged in the jejunum, the V/C ratio was significantly elevated in rTS-CAD_3_-treated pigeons (Figure 4C,D), which are closely linked to nutrient absorption efficiency and improved epithelial renewal. To further evaluate intestinal barrier function, occludin expression was analyzed by IHC and qPCR. Pronounced occludin immunostaining was observed in epithelial cells, predominantly in the upper regions of the villi in the rTS-CAD_3_ group (black arrows). rTS-CAD_3_ markedly increased occludin protein and mRNA levels in the duodenum and jejunum compared with the control groups (Figure 4E–G). Together, these results indicate that rTS-CAD_3_ improves intestinal morphology and strengthens barrier function, which may contribute to the reduced diarrhea incidence and improved growth performance observed in newborn pigeons.

## 4. Discussion

Safety remains an important consideration for NDV-based vectors, since NDV pathogenicity and in vivo replication can be affected by viral virulence, tissue tropism, host age, and host immune status [10,11]. Previous studies have shown that lentogenic NDV strains are generally safe and have been widely used as vaccine vectors, but their safety still requires evaluation in the target host [7]. Highly virulent NDV strains can cause severe systemic disease, and excessive inflammatory responses have been associated with disease severity and mortality [12]. In our study, rTS-CAD_3_ infection did not cause mortality, and viral RNA was detected only transiently in respiratory and intestinal tissues. In addition, IL-6 and G-CSF responses remained low, suggesting limited inflammatory activation. These findings suggest that rTS-CAD_3_ is safe in newborn pigeons in experimental conditions.

Antibiotics have been widely used to control bacterial diseases in poultry. However, their improper use in food-producing animals has raised concerns about antimicrobial residues in edible products and the spread of antimicrobial resistance [13,14]. Bacterial diarrhea in young pigeons during the early growth phase frequently necessitates clinical intervention, such as using antibiotics [15]. In our study, no antibiotics were used and CAD was expressed through an NDV-based vector. rTS-CAD_3_ reduced intestinal *E. coli* abundance and diarrhea incidence in newborn pigeons, suggesting that NDV-expressed CAD may help limit intestinal *E. coli* overgrowth. Therefore, this strategy may provide a non-antibiotic approach for enteric disease control in pigeon production.

CAD is a hybrid antimicrobial peptide composed of cecropin A and D domains. Previous studies have reported that dietary CAD supplementation can reduce *E. coli*-induced diarrhea and improve intestinal morphology and barrier function in piglets [4]. For poultry production, antimicrobial peptides are more practical when delivered through feed or drinking water; by contrast, injection or local administration is difficult to implement as a routine flock-level measure [16,17]. However, direct peptide supplementation is constrained by production and purification costs, limited gastrointestinal stability, proteolytic degradation, and repeated dosing requirements [18,19]. Here, rTS-CAD_3_ provides a vector-based delivery approach in which CAD is produced in vivo after intranasal administration of the NDV vector, rather than being repeatedly supplied as an exogenous peptide. However, rTS-CAD_3_ is a potential biological strategy that requires further validation, including spray delivery optimization, dose evaluation, and so on.

Previous studies have shown that antimicrobial peptides can improve intestinal health not only by directly inhibiting enteric pathogens, but also by modulating gut microbiota and strengthening epithelial barrier function [20,21]. Consistent with previous reports on dietary CAD, the present study showed that rTS-CAD_3_ reduced intestinal *E. coli* abundance and decreased the relative abundance of Escherichia–Shigella in newborn pigeons. The increase in Lactobacillus may enhance intestinal barrier health and microbial homeostasis by producing organic acids and suppressing opportunistic enteric pathogens, thereby contributing to improved gut health [22]. rTS-CAD_3_ also increased Firmicutes, improved villus height and V/C ratio, and upregulated occludin expression, a tight-junction-associated protein involved in maintaining epithelial structure. These findings suggest improved intestinal morphology and tight-junction-associated changes [23]. These changes were accompanied by increased ADG and reduced diarrhea incidence. Although the microbiome analysis was based on three samples per group and should therefore be considered exploratory, the results showed that the NDV vector may represent a practical strategy for transient in vivo delivery of CAD, potentially preserving some intestinal benefits without requiring continuous dietary supplementation, although the duration and tissue distribution of CAD expression remain to be determined.

In summary, rTS-CAD_3_ showed good safety in newborn pigeons under the experimental conditions and was associated with improved growth performance, reduced diarrhea, lower intestinal *E. coli* abundance, altered microbial composition, improved intestinal morphology, and increased occludin expression. Overall, rTS-CAD3-expressed CAD is beneficial for reducing intestinal *E. coli* abundance, improving gut health, and supporting growth performance in pigeon production.

## 5. Conclusions

In experimental conditions, rTS-CAD_3_ was safe for newborn pigeons and improved growth performance while reducing diarrhea. These beneficial effects were associated with reduced intestinal *E. coli* abundance, altered gut microbiota, improved intestinal morphology, and improved barrier health. Our findings suggest that NDV-based vectors may serve as a promising platform for antimicrobial peptide delivery and intestinal health improvement in poultry production.

## Figures and Tables

**Table 1:** EID_50_ and MDT values of rTS-CAD_3_ and TS09-C.

Strains	EID_50_/mL	MDT (h)
rTS-CAD_3_	10^9.0^	>168
TS09-C	10^8.5^	>168

## Data Availability

The 16S rRNA gene sequencing data generated in this study have been deposited in the NCBI Sequence Read Archive under BioProject accession number PRJNA1483584, with BioSample accession numbers SAMN61184894–SAMN61184902.

## Abbreviations

The following abbreviations are used in this manuscript:

CAD	cecropin AD
NDV	Newcastle disease virus
dpi	days post-infection
BW	body weight
ADG	average daily gain
G-CSF	granulocyte colony-stimulating factor
IL-6	Interleukin-6
*E. coli*	*Escherichia coli*
IHC	Immunohistochemistry
IOD	integrated optical density
